# The Protective Efficacy of an Inactivated Vaccine against *Avibacterium paragallinarum* Field Isolates

**DOI:** 10.3390/vetsci9090458

**Published:** 2022-08-26

**Authors:** Mengjiao Guo, Donghui Liu, Hengli Xu, Hao Zhang, Yikun Jin, Huihui Tan, Yantao Wu, Xiaorong Zhang

**Affiliations:** 1 Jiangsu Co-Innovation Center for Prevention of Animal Infectious Diseases and Zoonoses, College of Veterinary Medicine, Yangzhou University, Yangzhou 225009, China; 2 Joint International Research Laboratory of Agriculture & Agri-Product Safety, Yangzhou University (JIRLAAPS), Yangzhou 225009, China

**Keywords:** infectious coryza, *Avibacterium paragallinarum*, vaccine, protective efficacy

## Abstract

**Simple Summary:**

The incidence of infectious coryza has increased in China, even occurring in vaccinated chickens, causing serious economic losses. Several researchers believe that mutation, the increased virulence of *Avibacterium paragallinarum,* and improper vaccination procedures lead to poor protection from an inactivated vaccine. Based on this, the purpose of the current study was to evaluate the protective efficacy of a commercial infectious coryza trivalent inactivated vaccine against field infection of *Avibacterium paragallinarum* in China. Our results suggested that double vaccination showed better protection efficacy than single vaccination. The clinical symptoms, pathological changes, and bacterial shedding of chickens with double vaccination were significantly lower than those of control groups after challenge with field *Avibacterium paragallinarum* isolates of three serovars. No significant differences were found in body weight and egg production between the boost vaccination groups and the negative control group. The current commercial vaccine shows comparable efficacy in shortening the course of infection if administered twice. Our results provide a reference for infectious coryza clinical vaccine application in China. In addition, better ventilation strategies and biosecurity are important components of a strategic plan to prevent infectious coryza.

**Abstract:**

Infectious coryza (IC) is an acute respiratory disease caused by *Avibacterium paragallinarum* (*Av. paragallinarum*). In recent years, there have been frequent outbreaks of IC in chickens vaccinated with an inactivated vaccine, causing huge losses to the poultry industry. In this study, the protective efficacy of the trivalent inactivated IC vaccine (PT Medion Farma Jaya) against the field isolates of three serovars of *Av. paragallinarum* was verified. After vaccination, the hemagglutination inhibition antibody titers in double-vaccinated groups (A2, B2, and C2) were higher than those in single-vaccinated groups (A1, B1, and C1). The highest antibody titer was 2^13.1^ at 3 weeks after the booster vaccination in group A2. Consistent with the trend in hemagglutination inhibition antibody titers, the protective efficacy of double vaccination was better than that of single vaccination. The clinical symptoms and pathological changes were alleviated, or the bacterial shedding was significantly reduced with double vaccination after challenge with field isolates of three serovars (*p* < 0.05). In particular, the chickens with double vaccination showed no clinical symptoms, pathological changes, or bacterial shedding after challenge by the serovar C strain. There was no significant difference in body weight and egg production between the double-vaccinated groups and the negative control group (*p* > 0.05). Therefore, we recommend that the commercial IC vaccine should be double-vaccinated in clinical applications.

## 1. Introduction

Infectious coryza (IC) is an acute respiratory tract disease in chickens that is caused by *Avibacterium paragallinarum* (*Av. paragallinarum*), previously named *Haemophilus paragallinarum* [1,2]. IC is characterized by nasal discharge, facial swelling, and conjunctivitis, and it causes significant economic losses in the global poultry industry due to growth retardation in growing chickens and a marked reduction in egg production in layers, particularly on multi-aged farms. In broilers, a high condemnation rate caused by airsacculitis is an important outcome of IC. Another significant cost is incurred by the intensive vaccination protocol needed to control the disease [3]. IC is complicated by coinfection with bacteria such as *Mycoplasma gallisepticum*, *Mycoplasma synoviae*, *Gallibacterium anatis* [4], *Escherichia coli* [5], and *Ornithobacterium rhinotracheale* [6], and viruses that include infectious bronchitis virus, infectious laryngotracheitis virus, and fowl pox virus [7]. IC has occurred worldwide in recent years, including in the USA [8], Indonesia [9], the UK [10], and India [11]. In China, IC outbreaks have been reported in Beijing, Hebei, Shandong, Guangdong, Anhui, and Jiangsu provinces since 2012 [12,13]. Because of the limited use of antibiotics, bacterial diseases such as IC pose an increasingly serious threat to the poultry industry.

*Av. paragallinarum* was classified into three serovars (A, B, and C) by Page using agglutination [14,15]. Kume created an alternative scheme based on a hemagglutination inhibition (HI) test [16]. The modified Kume scheme recognizes serogroups A, B, and C, which match the Page serovars of A, B, and C, with nine serovars (A1–A4, B–1, and C1–C4) recognized [17]. The three Page serovars (A, B, and C) are generally considered to represent distinct immune variants [18]. In serogroup A, good cross-protection is prevalent among the four serotypes. In serogroup C, there is lower cross-protection within the four serotypes [19]. Although there is only one Kume serogroup B, only partial cross-protection has been detected between serotype B strains [20,21,22]. Three serotypes have been isolated in China. Serovar A was first reported in 1987, serovar C was first reported in 1995, and serovar B was first reported in 2003. In the past, monovalent (A) or bivalent (A and C) inactivated IC vaccines were often used in poultry farms. Currently, inactivated trivalent IC vaccines (A, B, and C) are mainly used in poultry farms. Recently, IC occurrence has increased in China, and the disease has even broken out in chickens immunized with bivalent or trivalent inactivated vaccines, indicating that some cases are associated with vaccine failure [23].

There are different perspectives on the reasons for vaccine failure. Some researchers have suggested that the current commercial vaccines do not provide sufficient protection. It is generally believed that there is no cross-protection among the Page serovars. Several researchers believed that variation and the increased virulence of *Av. paragallinarum* lead to poor protection within the same serovar, which would explain the lack of protection from the inactivated vaccine [23]. Some views propose that it is caused by improper vaccination procedures. Gallardo tested the effects of two commercially available vaccines on isolates in the United States. The experiment revealed that the commercial vaccines reduced clinical signs and bacterial shedding with double vaccination [7]. Based on this, the purpose of the current study was to investigate the protective efficacy of a commercial trivalent inactivated IC vaccine (PT Medion Farma Jaya) against field infection of *Av. paragallinarum* in China. Specifically, the two vaccination procedures (single vaccination and double vaccination) were conducted to evaluate the protection offered by the commercial IC vaccine.

## 2. Materials and Methods

### 2.1. Bacteria and Media

*Av. paragallinarum* isolates 2019−HB65 (serovar A), 2019−HB68 (serovar B), and 2020−JS80 (serovar C) isolated from chickens with clinical signs of IC were used as challenge strains [13]. Tryptic soy agar and tryptic soy broth, both supplemented with 10% fetal bovine serum (Thermo Fisher Scientific, Massachusetts, USA) and 0.0025% nicotinamide adenine dinucleotide (Solarbio, Beijing, China), were used to culture these isolates. The plates were cultured at 37 °C under 5% CO_2_ for 24–36 h. The typical colonies were inoculated into tryptic soy broth and cultured at 37 °C in a shaker for 16 h. According to the results of the viable count, the three bacterial suspensions were adjusted separately to 10^7^ CFU/mL (0.2 mL per chicken) for the challenge experiment.

### 2.2. Vaccine

The commercial trivalent inactivated IC vaccine was purchased from PT Medion Farma Jaya. The vaccine contained at least *Av. paragallinarum* strain W 1 × 10^8^ CFU/mL, strain Spross 1 × 10^8^ CFU/mL, and strain Modesto 1 × 10^8^ CFU/mL.

### 2.3. Immunization Procedures

A total of 100 commercial Hy-line brown layers (50 days old) not vaccinated against IC were purchased from a chicken farm in Yangzhou, Jiangsu. All chickens were of similar weight. In order to confirm that none of these chickens were infected with *Av. paragallinarum*, throat swabs were collected from all experimental chickens for bacterial isolation and HPG-2 PCR, which is specific to *Av. paragallinarum* [24]. All chickens were randomly divided into 10 groups (10 chickens per group): 3 single-vaccinated groups (A1, B1, and C1), 3 double-vaccinated groups (A2, B2, and C2), 3 PBS groups (A0, B0, and C0), and 1 negative control group (D). Chickens in groups A1, B1, and C1 were injected with 0.5 mL PBS at 53 days old and 0.5 mL IC vaccine at 110 days old via intramuscular injection. Chickens in groups A2, B2, and C2 were injected with 0.5 mL IC vaccine at 53 days old and 110 days old, respectively. Chickens in groups A0, B0, C0, and D were injected with 0.5 mL PBS at 53 days old and 110 days old, respectively (Table 1). In this experiment, each group of chickens was housed in a separate negative pressure isolator, with adequate feed and water.

### 2.4. Antibody Titers Detected by Hemagglutination Inhibition Test

Before and after vaccination, blood was collected weekly from the wing veins of the 10 chickens of each group for the HI test, lasting until 11 weeks after the prime vaccination. The sera were pre-treated by adsorption with glutaraldehyde-fixed chicken erythrocytes to avoid nonspecific reactions. Briefly, the sera were diluted 5 times with 1% glutaraldehyde-fixed chicken erythrocytes and incubated at 37 °C for 2 h, then overnight at 4 °C. Antigens of *Av. paragallinarum* were prepared from freshly cultured bacteria. The bacteria except strain 2019−HB65 were treated with 10 U/mL hyaluronidase at 37 °C for 2 h. The 4-unit antigens were prepared according to hemagglutinin (HA) titer. The HI tests were performed by double diluting sera with PBS. The diluted sera were mixed with an equal volume of 4-unit HA antigens and incubated at 37 °C for 40 min. Then, 1% (*v*/*v*) glutaraldehyde-fixed chicken erythrocytes was added and incubated at 37 °C for 60 min. The maximum serum dilution that completely inhibited hemagglutination was considered to be the HI titer.

### 2.5. Challenge Infection Experiments

At 3 weeks after the booster vaccination, the chickens (now 130 days old) in groups A1, A2, and A0 were challenged by infraorbital sinus inoculation with 0.2 mL bacterial suspension of the 2019−HB65 strain (serovar A) containing 10^7^ CFU/mL. Chickens in groups B1, B2, and B0 were challenged with 0.2 mL bacterial suspension of the 2019−HB68 strain (serovar B) containing 10^7^ CFU/mL. Chickens in groups C1, C2, and C0 were challenged with 0.2 mL bacterial suspension of the 2020−JS80 strain (serovar C) containing 10^7^ CFU/mL. The chickens in negative control group D were inoculated with 0.2 mL PBS in the same way (Table 1).

### 2.6. Clinical Scoring and Necropsy

After challenge, clinical signs were monitored daily. Briefly, the degrees of nasal discharge and facial swelling were scored at 4 levels as follows: 0, no clinical signs; 1, slight nasal discharge or facial swelling; 2, moderate nasal discharge and facial swelling; and 3, severe nasal discharge, facial swelling, and lacrimation [25]. The protective effect of the vaccine was evaluated by noting whether the vaccinated chickens had clinical symptoms of nasal discharge or facial swelling [26]. The chickens’ individual body weights were measured before and after challenge. The egg production was counted until 14 days post-challenge (dpc). Five chickens from each group were randomly selected and euthanized at 7 dpc. The gross lesions focused on infraorbital sinuses and tracheas were recorded during necropsy.

### 2.7. Av. paragallinarum Quantification by Quantitative Real-Time PCR

To evaluate the bacterial shedding, nasal swab samples were collected from each chicken at 3, 7, and 12 dpc. DNA was extracted by using the DNA extraction Kit (Cwbio, Beijing, China) according to the manufacturer’s instructions. The primers for detection of *Av. paragallinarum* were synthetized by Genscript Biotech Corporation (Nanjing, China), as described by Wen et al. [27]. Forward primer, 5′-AGCTTGCTCTACCGCACAAT-3′; Reward primer, 5′-CTGGCTTCTTGCACCTGAAT-3′. The quantitative real-time PCR (qRT-PCR) was performed using TransStartR Tip Green qPCR SuperMix (Transgen Biotech Co., Ltd., Beijing, China). The procedure for qRT-PCR: 94 °C for 30 s; followed by 40 circles of 94 °C 5 s; 60 °C 34 s. Finally, the dissociation curve analysis was performed.

### 2.8. Statistical Analysis

SPSS version 23.0 (SPSS Inc., Chicago, IL, USA) was used to analyze statistical data. The non-parametric Mann–Whitney U test was used to analyze the significant difference in different groups, and differences were considered significant at *p* < 0.05.

## 3. Results

### 3.1. Antibody Titers Detected by Hemagglutination Inhibition Test

**Figure 1:**
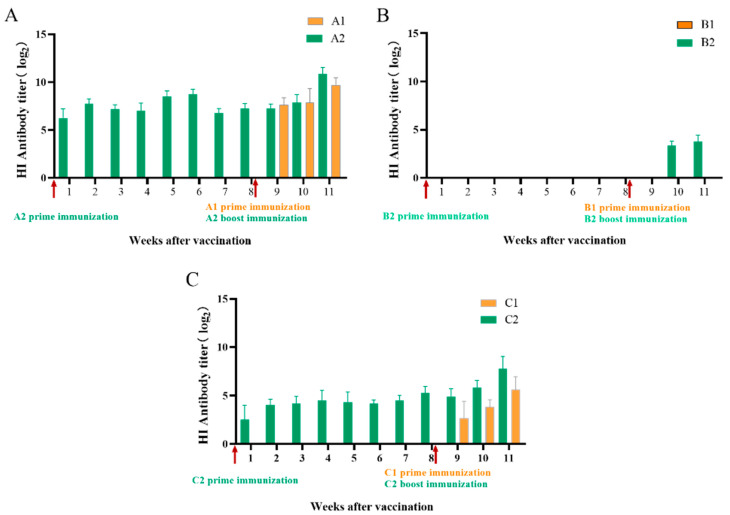
Hemagglutination inhibition antibody titers in chickens after prime and booster immunization. Serum samples (n = 10) were collected every week after vaccination until 11 weeks. Antibody titers were detected by HI test with 4 HA units prepared by the 2019−HB65 (**A**), 2019−HB68 (**B**), and 2020−JS80 (**C**). The antibody titers were expressed in the log_2_ form. Bars indicate means ± standard deviations.

Based on the bacterial testing of the throat swabs, no chickens were previously infected with *Av. paragallinarum*. The chickens in groups A1, B1, and C1 were vaccinated with the IC vaccine at 110 days old. In groups A2, B2, and C2, prime immunization was given at 53 days old and boosted at 110 days old. No HI antibodies to *Av. paragallinarum* were detected before vaccination. For group A1 and group A2, antibodies could be detected after the prime vaccination. The antibody titers increased after the booster vaccination in group A2. The highest antibody titer was 2^13.1^ at 3 weeks after the booster vaccination (Figure 1A). In contrast, no HI antibody was detected in groups B1 and B2 after the prime vaccination, and low levels of HI antibody were detected at 2 weeks after the booster vaccination (Figure 1B). The trend of antibody titers in groups C1 and C2 was similar to that in groups A1 and A2. The highest antibody titer was 2^10.2^ at 3 weeks after the booster vaccination in group C2 (Figure 1C).

### 3.2. Clinical Sign Score

**Figure 2:**
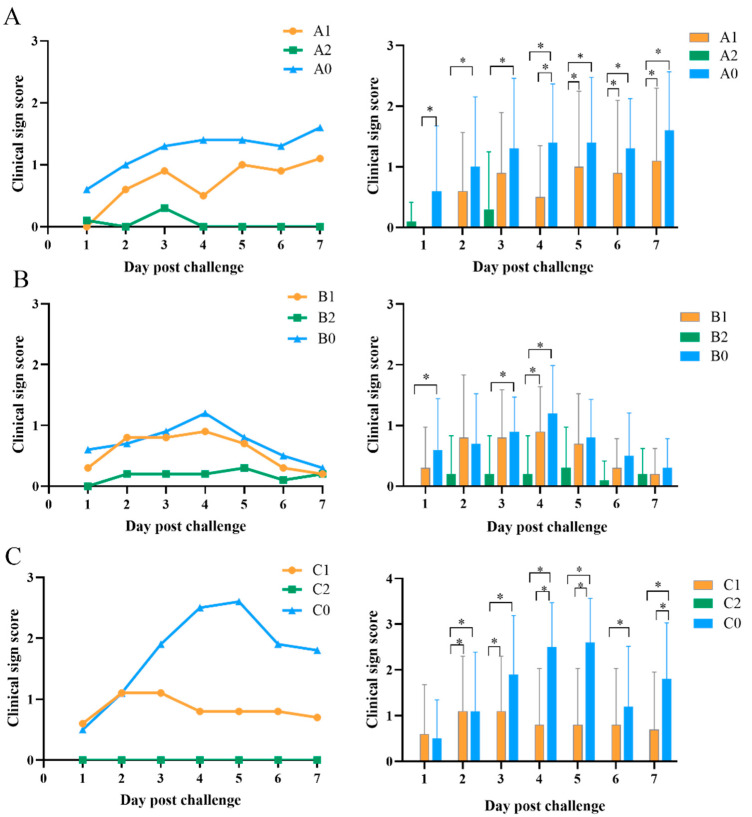
Clinical sign scores of chickens after challenge with *Av. paragallinarum.* Clinical signs scores (n = 10) were monitored daily for 7 days after challenge with 2019−HB65 (**A**), 2019−HB68 (**B**), and 2020−JS80 (**C**) at 130 days old. Data in line graphs are presented as means. Data in bar graphs indicate means ± standard deviations. * *p* < 0.05.

The clinical sign scores of the chickens after challenge are depicted in Figure 2. Typical clinical symptoms of IC, such as facial swelling and nasal discharge, appeared in the PBS-treated groups (A0, B0, and C0) after 1 dpc. The morbidity rate reached 80–90%, but no chickens died. The clinical symptoms in the three PBS groups were more serious than in the vaccination groups after challenge. The clinical sign scores of chickens with double vaccination were lower than those with single vaccination. Clinical sign scores of the chickens in group A0 increased continuously for 1–7 days after challenge. Clinical sign scores of the chickens in group A1 were lower than those in group A0. The chickens in group A2 showed only slight clinical symptoms at 1 dpc and 3 dpc and no symptoms after 4 dpc. The clinical sign scores of the chickens in group A2 were significantly lower than those in groups A0 and A1 (*p* < 0.05; Figure 2A). There was no significant difference in clinical sign scores between groups B1 and B0 (*p* > 0.05), while clinical symptoms of the chickens in group B2 were significantly milder than those in group B0 (*p* < 0.05; Figure 2B). The clinical sign scores of the chickens in group C1 were significantly lower than those in group C0 (*p* < 0.05). Most importantly, no clinical symptoms were observed in the chickens in group C2 (Figure 2C).

### 3.3. Body Weight

**Figure 3:**
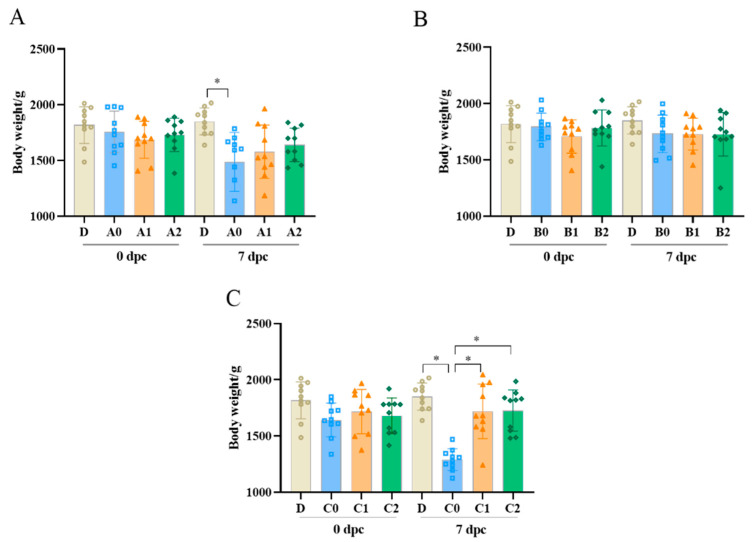
Body weight of chickens after challenge with *Av. paragallinarum.* The chickens’ individual body weights were measured before and 7 days after challenge with 2019-HB65 (**A**), 2019-HB68 (**B**), and 2020-JS80 (**C**) at 130 days old (n = 10). Data in bar graphs indicate means ± standard deviations. * *p* < 0.05.

As shown in Figure 3, the body weight gain of chickens in control group D was normal. The body weights of chickens in groups A0 and C0 were significantly lower than those in group D at 7 dpc (*p* < 0.05; Figure 3A,C). As shown in Figure 3A, the body weights of chickens in groups A1 and A2 decreased, but the difference was not significant. No significant differences in body weight were found between B0, B1, B2, and D (*p* > 0.05; Figure 3B). After challenge, the chickens in groups C1 and C2 did not lose weight (Figure 3C).

### 3.4. Egg Production

As shown in Table 2, the egg production of groups A0 and C0 was significantly lower than that of group D after challenge (*p* < 0.05). For the single-vaccinated chickens, there was a significant difference in the egg production rates between group A1 and group D after challenge (*p* < 0.05). In contrast, no significant differences in egg production was found between the boost immunized groups (A2, B2, and C2) and group D (*p* > 0.05).

### 3.5. Pathological Changes of Infraorbital Sinus and Trachea

**Figure 4:**
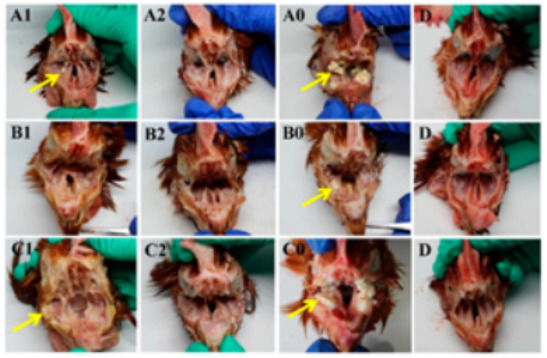
Gross lesions of the infraorbital sinus after challenge with *Av. paragallinarum*. The letters in the figure correspond to the groups. Yellow exudate clot (yellow arrows) was observed in the infraorbital sinus of chickens in groups A1, A0, B0, C1, and C0.

**Figure 5:**
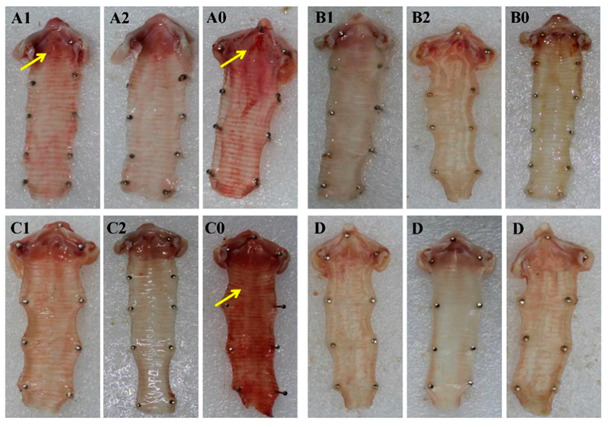
Gross lesions of the trachea after challenge with *Av. paragallinarum*. The letters in the figure correspond to the groups. Hemorrhage (yellow arrows) was observed in the trachea of chickens in groups A1, A0, and C0.

Five chickens from each group were randomly selected and euthanized at 7 dpc to observe pathological changes. No pathological changes were observed in the negative control group D. The severity of pathological changes in each group after challenge was consistent with clinical symptoms. Pathological changes were mainly observed in the infraorbital sinus and trachea. The severity of pathological changes in groups A0, B0, and C0 was more serious than that in the vaccinated groups. A large amount of yellow exudate clot was observed in the infraorbital sinus in chickens in groups A0, B0, and C0. A few caseous substances were observed in the infraorbital sinus in groups A1 and C1. However, no pathological changes were observed in groups A2, B2, and C2 at 7 dpc (Figure 4). As shown in Figure 5, a severe hemorrhage in the trachea was observed in groups A0 and C0, while minor hemorrhage was observed in group A1. No pathological changes in the trachea were observed in groups A2, B2, and C2 at 7 dpc. Thus, the pathological changes in chickens with double vaccination were milder than those with single vaccination.

### 3.6. Bacterial Shedding Quantification

**Figure 6:**
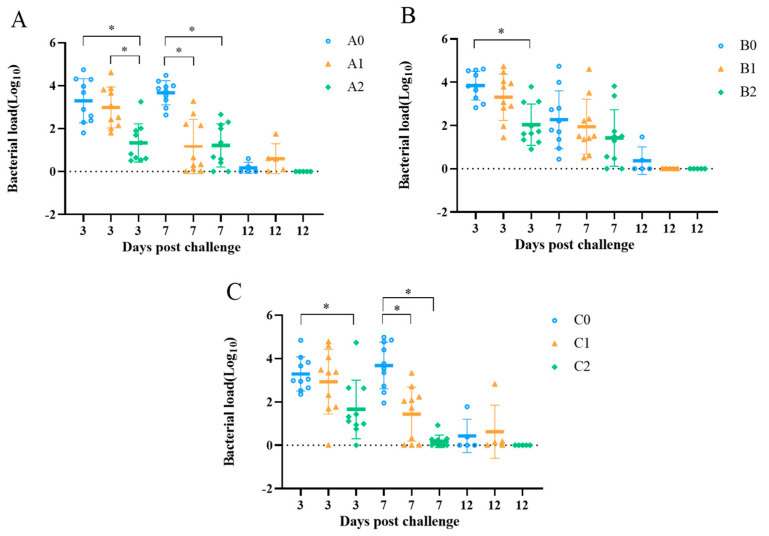
Bacterial shedding in chickens after challenge with *Av. paragallinarum.* The nasal swab samples were collected at 3, 7, and 12 days after challenge with 2019−HB65 (**A**), 2019−HB68 (**B**), and 2020−JS80 (**C**) at 130 days old. * *p* < 0.05.

Nasal swab samples were collected at 3, 7, and 12 dpc for bacterial quantification. As shown in Figure 6, the bacterial shedding was reduced in all vaccinated groups. In particular, the bacterial shedding in the double-vaccinated groups A2, B2, and C2 was significantly lower than that in groups A0, B0, and C0 at 3 dpc, respectively (*p* < 0.05). At 7 dpc, the bacterial shedding in groups A1, A2, C1, and C2 was significantly reduced (*p* < 0.05; Figure 6A). At 12 dpc, no bacteria were detected in groups A2, B1, B2, and C2 (Figure 6).

## 4. Discussion

All growth-promoting antibiotics have been banned as feed additives in China since 2020. The antibiotics are strictly restricted, particularly during the laying period. Thus, strict biosafety and vaccination protocols are the main measures to prevent IC. At present, trivalent inactivated vaccines have been widely used in many intensive chicken farms in China. However, IC has occurred even in vaccinated chickens. This suggests that the existing control measures are still unable to protect chickens from *Av. paragallinarum*. An in vivo vaccine efficacy experiment is an important method for evaluating the protective effect of an *Av. paragallinarum* vaccine on chickens. Therefore, in this study, we evaluated the protective efficacy of a commercial trivalent IC vaccine against field isolates of three serovars of *Av. paragallinarum*.

Many studies have shown that the HA antigen plays an important role in the pathogenicity and immunogenicity of *Av. paragallinarum* [28,29,30]. In this study, we found that the HI antibody titers in groups A and C were higher than that in group B and that these HI titers increased after booster vaccination in all groups. Although several other studies have suggested that high antibody levels are not considered a decisive predictor of IC vaccine efficacy [31,32]. Our results showed that the HI antibody titers were positively correlated with the protective efficacy of the IC vaccine. After challenge with *Av. paragallinarum*, the protective effect of double vaccination was better than that of single vaccination. This result indicated that the level of HI antibody titer can be used to evaluate the efficacy of the IC vaccine.

It is generally recognized that the IC vaccine is protective against *Av. paragallinarum* when the serovars are matched. According to the cross-protection study conducted with the nine Kume serovars, cross-protection was only observed between C−4 and B−1 [19]. It has been suggested that some cases of immune failures of IC vaccines may be from the lack of cross-protection between vaccine strains and field isolates. Therefore, the *Av. paragallinarum* local isolates 2019−HB65, 2019−HB68, and 2020−JS80 were used for this challenge experiment. Vaccine manufacturers recommend single and double vaccination procedures, but due to considerations of cost, management, and adverse reactions such as stress, farmers sometimes adopt the single vaccination procedure. This is believed to be one of the reasons for the failure of vaccination; consequently, this study also focused on the evaluation of single vaccination and double vaccination procedures. Our results showed that the clinical symptoms and pathological changes of vaccinated chickens were alleviated after challenge. The clinical sign scores in chickens with double vaccination were lower than those with single vaccination. Most importantly, the chickens with double vaccination in group C2 had no clinical symptoms after challenge. This result was consistent with a previous study [33]. At 7 dpc, no pathological changes in the infraorbital sinus and trachea were observed in the three double-vaccinated groups. In addition, no significant differences in body weight and egg production were found between the double-vaccinated groups after challenge and the negative control group. Bacterial shedding reflects the risk of IC transmission in chickens. In this study, the shedding of *Av. paragallinarum* in double-vaccinated groups was lower than that in the PBS groups after challenge. Our results confirmed that single vaccination with a commercial IC vaccine provides some protection against field strains and shortens the period of *Av. paragallinarum* infection, but the efficacy of a single vaccination is not sufficiently high. In contrast, the double-vaccinated chickens were completely protected against the field serovar C isolate as well as partially protected against the field serovar A and B isolates. Therefore, replacing the serotype A and B strains in existing vaccines with local isolates would be a better option for future vaccines.

In field cases, IC clinical symptoms are complicated by coinfection with concomitant respiratory pathogens in commercial flocks. The presence of these pathogens may lead to complex clinical diseases, increasing septicemia and mortality [7,10]. Poor management and ventilation can promote complex respiratory diseases [34,35,36]. Although no more-effective commercial IC vaccines are available on the market, the currently available vaccine shows comparable efficacy in reducing the clinical symptoms and shedding of *Av. paragallinarum* if administered twice, in line with the manufacturer’s recommendation. In addition to vaccination, better ventilation strategies and biosafety to minimize additional challenges are important parts of a good IC prevention strategic plan.

In conclusion, the efficacy of the vaccine is better in chickens with high HI antibody levels. The trivalent inactivated vaccine did not completely protect chickens against three serovars of *Av. paragallinarum* field isolates. However, the results showed that vaccination plays an important role in shortening the course of infection. Double vaccination showed better protection efficacy than single vaccination, and double vaccination provided protection efficacy related to weight and egg production after challenge. Our results provide a reference for clinical vaccine application in China.

## Figures and Tables

**Table 1:** Immunization procedure and challenge experiments design.

Groups	Number of Chickens	Times of Vaccination	Age of Vaccination	Strain for Challenge	Age of Challenge
A1	10	1	110 d	2019−HB65	130 d
B1	10	1	110 d	2019−HB68	130 d
C1	10	1	110 d	2020−JS80	130 d
A2	10	2	53 d and 110 d	2019−HB65	130 d
B2	10	2	53 d and 110 d	2019−HB68	130 d
C2	10	2	53 d and 110 d	2020−JS80	130 d
A0	10	/	/	2019−HB65	130 d
B0	10	/	/	2019−HB68	130 d
C0	10	/	/	2020−JS80	130 d
D	10	/	/	/	/

**Table 2:** Egg production rate after challenged with *Av. paragallinarum*.

Groups	7 dpc	14 dpc
A1	15.71 ± 12.94 ^a^	20.00 ± 15.12 ^b^
A2	28.57 ± 15.52	25.71 ± 20.60
A0	15.71 ± 12.94 ^a^	6.29 ± 8.78 ^b^
B1	30.00 ± 16.90	22.86 ± 12.78
B2	45.71 ± 11.78	22.86 ± 16.66
B0	32.86 ± 12.78	22.86 ± 7.00
C1	24.29 ± 10.50	24.29 ± 15.91
C2	30.00 ± 7.56	37.14 ± 16.66
C0	11.43 ± 9.90 ^a^	0.00 ± 0.00 ^b^
D	37.14 ± 13.85	34.29 ± 9.04

Different superscript lowercase letters indicate significant differences between challenge groups and negative control group D (*p* < 0.05).

## Data Availability

The data presented in this study are available in the article.

## References

[1] Blackall P.J. (1989). The avian haemophili. Clin. Microbiol. Rev..

[2] Blackall P.J., Christensen H., Beckenham T., Blackall L.L., Bisgaard M. (2005). Reclassification of Pasteurella gallinarum, [Haemophilus] paragallinarum, Pasteurella avium and Pasteurella volantium as Avibacterium gallinarum gen. nov., comb. nov., *Avibacterium paragallinarum* comb. nov., Avibacterium avium comb. nov. and Avibacterium volantium comb. nov. Int. J. Syst. Evol. Microbiol..

[3] Blackall P.J., Soriano-Vargas E. (2020). Infectious coryza and related bacterial infections. Diseases of Poultry.

[4] Paudel S., Hess M., Hess C. (2017). Coinfection of *Avibacterium paragallinarum* and Gallibacterium anatis in Specific-Pathogen-Free Chickens Complicates Clinical Signs of Infectious Coryza, Which Can Be Prevented by Vaccination. Avian Dis..

[5] Crispo M., Blackall P., Khan A., Shivaprasad H.L., Clothier K., Sentíes-Cué C.G., Cooper G., Blakey J., Pitesky M., Mountainspring G. (2019). Characterization of an Outbreak of Infectious Coryza (*Avibacterium paragallinarum*) in Commercial Chickens in Central California. Avian Dis..

[6] Morales-Erasto V., Falconi-Agapito F., Luna-Galaz G.A., Saravia L.E., Montalvan-Avalos A., Soriano-Vargas E.E., Fernández-Díaz M. (2016). Coinfection of *Avibacterium paragallinarum* and *Ornithobacterium rhinotracheale* in Chickens from Peru. Avian Dis..

[7] Gallardo R.A., Da Silva A.P., Egaña-Labrin S., Stoute S., Kern C., Zhou H., Cutler G., Corsiglia C. (2020). Infectious Coryza: Persistence, Genotyping, and Vaccine Testing. Avian Dis..

[8] Crispo M., Sentíes-Cué C.G., Cooper G.L., Mountainspring G., Corsiglia C., Bickford A.A., Stoute S.T. (2018). Otitis and meningoencephalitis associated with infectious coryza (*Avibacterium paragallinarum*) in commercial broiler chickens. J. Vet. Diagn. Investig..

[9] Wahyuni A., Tabbu C.R., Artanto S., Setiawan D.C.B., Rajaguguk S.I. (2018). Isolation, identification, and serotyping of *Avibacterium paragallinarum* from quails in Indonesia with typical infectious coryza disease symptoms. Vet. World.

[10] De Welchman D.B., King S.A., Wragg P., Wood A.M., Irvine R.M., Pepper W.J., Dijkman R., de Wit J.J. (2010). Infectious coryza in chickens in Great Britain. Vet. Rec..

[11] Patil V.V., Mishra D., Mane D.V. (2017). 16S ribosomal RNA sequencing and molecular serotyping of *Avibacterium paragallinarum* isolated from Indian field conditions. Vet. World.

[12] Sun H., Xie S., Li X., Xu F., Li Y., Boucher C.E., Chen X. (2018). Selection of *Avibacterium paragallinarum* Page serovar B strains for an infectious coryza vaccine. Vet. Immunol. Immunopathol..

[13] Guo M., Chen X., Zhang H., Liu D., Wu Y., Zhang X. (2022). Isolation, Serovar Identification, and Antimicrobial Susceptibility of *Avibacterium paragallinarum* from Chickens in China from 2019 to 2020. Vet. Sci..

[14] Page L.A. (1962). Haemophilus infections in chickens. I. Characteristics of 12 Haemophilus isolates recovered from diseased chickens. Am. J. Vet. Res..

[15] Page L.A., Rosenwald A.S., Price F.C. (1963). Haemophilus infections in chickens. IV. Results of laboratory and fieldtrials of formalinized bacterins for the prevention of disease caused by *Haemophilus gallinarum*. Avian Dis..

[16] Kume K., Sawata A., Nakai T., Matsumoto M. (1983). Serological classification of *Haemophilus paragallinarum* with a hemagglutinin system. J. Clin. Microbiol..

[17] Blackall P.J., Eaves L.E., Rogers D.G. (1990). Proposal of a new serovar and altered nomenclature for *Haemophilus paragallinarum* in the Kume hemagglutinin scheme. J. Clin. Microbiol..

[18] Blackall P.J. (1999). Infectious coryza: Overview of the disease and new diagnostic options. Clin. Microbiol. Rev..

[19] Soriano E.V., Garduño M.L., Téllez G., Rosas P.F., Suárez-Güemes F., Blackall P.J. (2004). Cross-protection study of the nine serovars of *Haemophilus paragallinarum* in the Kume haemagglutinin scheme. Avian Pathol..

[20] Yamaguchi T., Blackall P.J., Takigami S., Iritani Y., Hayashi Y. (1991). Immunogenicity of *Haemophilus paragallinarum* serovar B strains. Avian Dis..

[21] Jacobs A.A., van den Berg K., Malo A. (2003). Efficacy of a new tetravalent coryza vaccine against emerging variant type B strains. Avian Pathol..

[22] Morales-Erasto V., Fernández-Rosas P., Negrete-Abascal E., Salazar-García F., Blackall P.J., Soriano-Vargas E. (2014). Genotyping, pathogenicity, and immunogenicity of *Avibacterium paragallinarum* serovar B-1 isolates from the Americas. Avian Dis..

[23] Xu Y., Cheng J., Huang X., Xu M., Feng J., Liu C., Zhang G. (2019). Characterization of emergent *Avibacterium paragallinarum* strains and the protection conferred by infectious coryza vaccines against them in China. Poult. Sci..

[24] Chen X., Miflin J.K., Zhang P., Blackall P.J. (1996). Development and application of DNA probes and PCR tests for *Haemophilus paragallinarum*. Avian Dis..

[25] Bragg R. (2002). Virulence of South African isolates of *Haemophilus paragallinarum*. Part 1: NAD-dependent field isolates. Onderstepoort J. Vet. Res..

[26] Gong Y., Zhang P., Wang H., Zhu W., Sun H., He Y., Shao Q., Blackall P.J. (2014). Safety and efficacy studies on trivalent inactivated vaccines against infectious coryza. Vet. Immunol. Immunopathol..

[27] Wen S., Chen X., Xu F., Sun H. (2016). Validation of Reference Genes for Real-Time Quantitative PCR (qPCR) Analysis of *Avibacterium paragallinarum*. PLoS ONE.

[28] Kume K., Sawata A., Nakase Y. (1980). Relationship between protective activity and antigen structure of *Haemophilus paragallinarum* serotypes 1 and 2. Am. J. Vet. Res..

[29] Takagi M., Hirayama N., Makie H., Ohta S. (1991). Production, characterization and protective effect of monoclonal antibodies to *Haemophilus paragallinarum* serotype A. Vet. Microbiol..

[30] Morales-Erasto V., Maruri-Esteban E., Trujillo-Ruíz H.H., Talavera-Rojas M., Blackall P.J., Soriano-Vargas E. (2015). Protection Conferred by Infectious Coryza Vaccines Against Emergent *Avibacterium paragallinarum* Serovar C-1. Avian Dis..

[31] García A., Romo F., Ortiz A.M., Blackall P.J. (2008). The vaccination-challenge trial: The gold standard test to evaluate the protective efficacy of infectious coryza vaccines. Avian Pathol..

[32] Sakamoto R., Baba S., Ushijima T., Kino Y., Honda T., Mizokami H., Sakaguchi M. (2013). Development of a recombinant vaccine against infectious coryza in chickens. Res. Vet. Sci..

[33] Han M.S., Kim J.N., Jeon E.O., Lee H.R., Koo B.S., Min K.C., Lee S.B., Bae Y.J., Mo J.S., Cho S.H. (2016). The current epidemiological status of infectious coryza and efficacy of PoulShot Coryza in specific pathogen-free chickens. J. Vet. Sci..

[34] Shepherd T.A., Zhao Y., Li H., Stinn J.P., Hayes M.D., Xin H. (2015). Environmental assessment of three egg production systems—Part II. Ammonia, greenhouse gas, and particulate matter emissions. Poult. Sci..

[35] Zhao Y., Zhao D., Ma H., Liu K., Atilgan A., Xin H. (2016). Environmental assessment of three egg production systems—Part III: Airborne bacteria concentrations and emissions. Poult. Sci..

[36] Zhao Y., Shepherd T.A., Li H., Xin H. (2015). Environmental assessment of three egg production system—Part I: Monitoring system and indoor air quality. Poult. Sci..

